# Comparison of total and *aqua regia* extractability of heavy metals in sewage sludge: The case study of a certified reference material

**DOI:** 10.1016/j.trac.2017.01.010

**Published:** 2017-04

**Authors:** Anna Santoro, Andrea Held, Thomas P.J. Linsinger, Andres Perez, Marina Ricci

**Affiliations:** European Commission Joint Research Centre, Directorate F – Health, Consumers and Reference Materials, Retieseweg 111, 2440 Geel, Belgium

**Keywords:** Sewage sludge, Soil contamination, Pollutant, Metals, ISO 12914, ISO 11466, Reference material

## Abstract

A number of different digestion methods, including *aqua regia* extraction following two ISO guides were used in an inter-laboratory comparison study. The results obtained showed comparable values for the total and *aqua regia* extractable content of As, Cu, Fe, Hg, Pb and Zn, while Cd, Co and Cr results were about 10% lower when *aqua regia* was employed. This small difference was covered by the between-laboratory relative standard deviation of the measurements; therefore in this study no difference in the extraction of the elements by the employed methods was found. The high organic matter content, together with low SiO_2_ and refractory aluminium and iron oxide amount as well as the small particle size of the sewage sludge material was reputed to have an effect on the extracting capacity of a weaker solvent such as *aqua regia*, bringing its results close to the total content ones.

## Highlights

• The use of sewage sludge as agricultural fertiliser is regulated in most countries and in particular in Europe by the Directive 86/278/EEC (1986).
• Total and *aqua regia* extractable content of 11 elements were determined in sewage sludge through an inter-laboratory comparison study.
• Total and *aqua regia* extractable content were comparable within the between-laboratory relative standard deviation of the measurements.
• The extractability of the elements related to the amount of refractory oxide, organic matter content and small particle size of the sample matrix.

## 1 Introduction

### 1.1 Historical background

The importance of accurately measuring heavy metals in soil, sediment and similar matrices, including sewage sludge materials, so as to better evaluate their potential ecotoxicity and further develop rigorous environmental policy, has long been researched [1], [2]. As pointed out by these studies, the results obtained could be strongly influenced by the methodology used or extraction protocol adopted (operationally-defined data), often making them difficult to compare. Method standardisation in order to harmonise measurement results was needed and extensive work was carried out during the '90s both thanks to the International Standard Organisation (ISO) and at European level under the Community Bureau of Reference (BCR) programme and its successor, the Standards, Measurement and Testing programme of the European Commission [3], [4]. In particular, these earlier studies have focused on the standardisation of widely used extraction methods based on inter-laboratory comparison exercises involving expert laboratories. The accuracy of the results obtained by such operationally-defined harmonised/standardised methods was then verified using carefully-prepared certified reference materials as quality control tools [5]. The work carried out within the BCR research framework, later merged into the activities of the Institute for Reference Materials and Measurements (IRMM), has been significant in this sense.

It is building on this standardisation process carried out over the past 20–25 years that the work presented here shows how it is still relevant to properly evaluate the analytical methodology when assessing the extractability of metals in environmental matrices with regard to the quality and comparability of the measurement results.

### 1.2 Current trends in sewage sludge determinations

Treated sewage sludge materials, either of industrial or domestic origin, have increasingly been used in the last decades on farmland, mostly for their fertilising properties, due to its high content of organic matter as well as of plant macro- and micro-nutrients [6], [7], [8]. Application of sewage sludge to agricultural land partially solves the problem of sewage sludge disposal after production, but on the other hand may pose risks related to the contamination of soil and ground water by inorganic and organic pollutants present in the sludge [9], [10]. In particular, heavy metals such as cadmium (Cd), nickel (Ni) and lead (Pb) once applied to soils may enter the food chain through plants and animals, contaminate surface and ground water, eventually causing hazards to human health [9], [11], [12]. Landfill of sewage sludge is regulated worldwide, with some countries imposing stricter limits than others [13], [14]. In Europe, the EU Directive 86/278/EEC [13] with its amendments from 1991 and 2009, regulates the use of sewage sludge in agriculture disposal and prohibits its use unless specific requirements are fulfilled, including compliance with limit values of a range of trace elements. Specific guidelines governing land application of sewage sludge are furthermore acted at local/national level.

In this respect, many laboratories perform a number of analyses to determine the content of trace elements in sewage sludge in order to evaluate their potential health and environmental risk. Mostly, laboratories determine the total content of trace elements performing a complete dissolution of the solid matrix with a combination of strong acids, often including hydrofluoric acid as well as different alkali solutions.

Some national regulations may however request the evaluation of the *aqua regia* extractable content of the elements rather than the total one, sometimes called ‘pseudo-total’ content [3], as a good estimate of the bioavailable fraction of heavy metals [7], [15]. *Aqua regia* extractability of metals from sewage sludge and related materials is described in two International Standard Organisation (ISO) procedures. The ISO standard 11466 [16] describes the use of an assembled reflux digestion system with temperature-controlled heating apparatus and a substantial amount of *aqua regia* and sample. More recently, the ISO 12914 [17] specifies the use of microwave-assisted digestion of elements, with reduced amounts of the sample and *aqua regia*.

Although commonly used, *aqua regia* is often rated as a less suitable solvent for the estimation of the total amount of heavy metals in an environmental matrix. Past works have shown that *aqua regia* extraction may lead to an underestimation of the amount of metals such as Co, Cd, Cr and Ni by up to 50% [18], [19]. The reasons for this underestimation lies on the type of matrix and the strength of the solvent itself, which is unable to dissolve silicates and aluminium and iron oxides to which some metals may be bound. Nonetheless, Sastre et al. [20] demonstrated in their study that the discrepancy between total and *aqua regia* extractable content cannot be regarded as a general rule and may depend strongly on the element analysed, chemical composition of the matrix, mainly organic matter content, and type of solvents used for the total extraction.

This study presents the results obtained during the certification process of a sewage sludge reference material whose characterisation was carried out through an inter-laboratory comparison study, and focuses on the comparison between the total content and the *aqua regia* extractable content, determined using the ISO 11466 and ISO 12914 standards, of As, Cd, Co, Cr, Cu, Fe, Hg, Mn, Ni, Pb and Zn.

## 2 Materials and methods

### 2.1 Collection and characterisation of the sewage sludge

The sewage sludge used in this study was sampled at the waste water treatment plants (WWTP) of Amsterdam and surroundings. Once collected, the material was dried at room temperature, homogenised for about 30 min using a Turbula^®^ mixer at 22 rpm speed and finely ground to a particle size <250 μm using a multi-processing system (100 AFG Jet Mill, Alpine, Germany). The particle size analysis of the material was carried out on three separate sub-samples dispersed in methanol using a Helos laser light scattering instrument (Sympatec, DE). The total amount of ca. 94 kg of sewage sludge powder was divided into 30 g aliquots and placed in dark glass bottles, sealed with polyethylene insert and a screw cap. All samples were stored at 4°C throughout the whole study.

Water content was determined by volumetric Karl-Fisher titration and the water activity using a water activity meter (Aqualab CX3, Decagon, USA). Total carbon (TC) and total inorganic carbon (TIC) were determined using a combustion furnace (Ströhleim C-mat 5500, DE) following the ISO 10694 [21]. Total organic carbon (TOC) was obtained as difference between TC and TIC. Major components, such as Al, Ca, K, Mg and Na were determined by *k*_0_-NAA, while SiO_2_ and P_2_O_5_ were determined by gravimetry and spectrophotometry, respectively.

Homogeneity and stability (up to 60°C and 36 months) of the test material were checked before the inter-laboratory comparison study. The uncertainty contributions related to possible heterogeneity and instability were estimated to be <3% for all the elements, confirming the suitability of the selected material to be further characterised as a certified reference one.

### 2.2 Inter-comparison study setup

A set of technical criteria was used to select the laboratories participating in the inter-laboratory comparison study leading to the certification of the sewage sludge material. In particular, participating laboratories had to have a quality management system in place (either a formal ISO/IEC 17025 accreditation or conforming to it), so as to ensure that analyses' results were fully trustable, as well as experience and/or participation in previous inter-laboratory comparison studies. In order to randomise possible laboratory bias, a number of measures were taken: each selected laboratory provided independent measurements spread over two days to ensure within-laboratory intermediate precision conditions for each element and different methodologies for total content were employed, with a final total number of laboratories ≥6. Regarding the *aqua regia* extractable content, the ISO procedures were strictly followed. Each laboratory analysed two bottles of the sewage sludge material in triplicate (*n* = 3), for a total of six results corrected for water content and provided an estimation of the uncertainty related to their measurements, following their own approach.

In order to check the trueness of each laboratory's method and the absence of specific laboratory bias, two certified reference materials (CRM), BCR-144R (sewage sludge of domestic origin) and BCR-146R (sewage sludge of industrial origin) were given to the participants to be analysed as quality control samples. Results significantly deviating from the certified values of these two CRMs were considered affected by bias and thus not used in the final evaluation of the inter-laboratory comparison results.

### 2.3 Analytical methods

#### 2.3.1 Total element mass fractions

In order to determine the total mass fractions of the elements, nine laboratories (L1–L9) employed closed-microwave digestion using an amount of sample ranging from 0.1 g to 1 g and a combination of hydrofluoric acid (HF) and one or more of other digestion media such as hydrochloric acid (HCl), hydrogen peroxide (H_2_O_2_) and perchloric acid (HClO_4_). The analytical techniques used for the determination of the total amount of the elements in the extracts were mainly inductively coupled plasma, mass or optical emission spectrometry (ICP-MS and ICP-OES) and flame atomic absorption spectrometry (FAAS) for most of the elements. Cold vapour (CV) atomic absorption or fluorescence spectroscopy (AAS and AFS) and solid sample direct mercury analysis (DMA) were preferentially used for Hg.

The digestion-free technique neutron activation analysis (*k*_0_-NAA), recognised as having the potential of a primary ratio method [22], was also used by two participating laboratories (L10–L11) on solid samples for the determination of the total content of a range of elements. Samples were irradiated in two different reactors and gamma-ray counting was performed using a coaxial HPGe detector. The elements determined were (in parenthesis the isotope and the gamma-ray emissions): As (^76^As, 559.1 keV); Cd (^15^Cd, 336.2 keV); Co (^15^Co, 1173.2 keV, 1332.5 keV); Cr (^51^Cr, 320.1 keV); Cu (^64^Cu, 1345.8 keV); Hg (^203^Hg, 279.2 keV); Fe (^59^Fe, 1099.3 keV, 1291.6 keV); Mn (^56^Mn, 846.8 keV, 1810.7 keV, 2113.1 keV) and Zn (^65^Zn, 1115.5 keV). Extended details on the methods and techniques used during this inter-laboratory comparison study are presented in Table 1a, Table 1ba and 1b.

#### 2.3.2 *Aqua regia* extractable element content

The *aqua regia* extractable content of the elements was determined following two ISO standard procedures: ISO 11466:1995 [16] and ISO 12914:2012 [17]. Laboratories L12-L20 used ISO 11466:1995 and employed 3 g of sample and about 28 mL of *aqua regia* for the extraction, followed by filtration of the extract through ashless paper filters and dilution with deionised water. Laboratories L21–L26 used the ISO 12914:2012 which requires a minimum of 0.5 g of sample mixed with 8 mL of *aqua regia* and digested in a microwave digestion vessel at a temperature of 175°C for 20 min. The extracts were then filtered through ashless paper filters and diluted to need. Determination of the mass fraction was performed as reported in Table 1a, Table 1ba and 1b, mainly with spectroscopic techniques.

### 2.4 Uncertainty estimations and statistical evaluation of results

As there was no specific restriction in the estimation of the measurement uncertainties given by the EU directive [13] or ISO procedures employed, laboratories were free to choose their own approach. The measurement uncertainty was in most cases based on 1* or 2*σ, with σ being the relative standard deviation of measurements within laboratory; otherwise sometimes including a bias related to the digestion method (Table 1a, Table 1ba and 1b) and often applying a coverage factor of *k* = 2 [23].

All data received were first grouped according to the method used: total content with spectroscopic method (tc_1_), total content with *k*_0_-NAA (tc_2_), *aqua regia-*ISO11466 (ar_1_) and *aqua regia-*ISO12914 (ar_2_) and checked for normality of the distribution, any major bias or outliers.

A *t*-test was used to compare results according to the type of extraction, hence total content (tc_1_ and tc_2_) *vs aqua regia* extractable content (ar_1_ and ar_2_) as well as for combined data total content (tc) *vs aqua regia* (ar).

## 3 Results and discussion

### 3.1 Total content

The results obtained for the total content of the elements determined using microwave digestion and atomic (absorption or emission) spectroscopy by laboratories L1–L9 (tc_1_) as well as the results obtained from L10 and L11 with *k*_*0*_-NAA (tc_2_) were all normally distributed and no outliers were observed. In a few cases, results for a specific element and/or laboratory were eliminated on the basis of disagreement between the measured and the certified value of the quality control sample. This technical evaluation was carried out to flag and possibly exclude results related to poor laboratory performance for that specific element or extraction method, which inclusion could bias the outcome of the study.

**Fig. 1:**
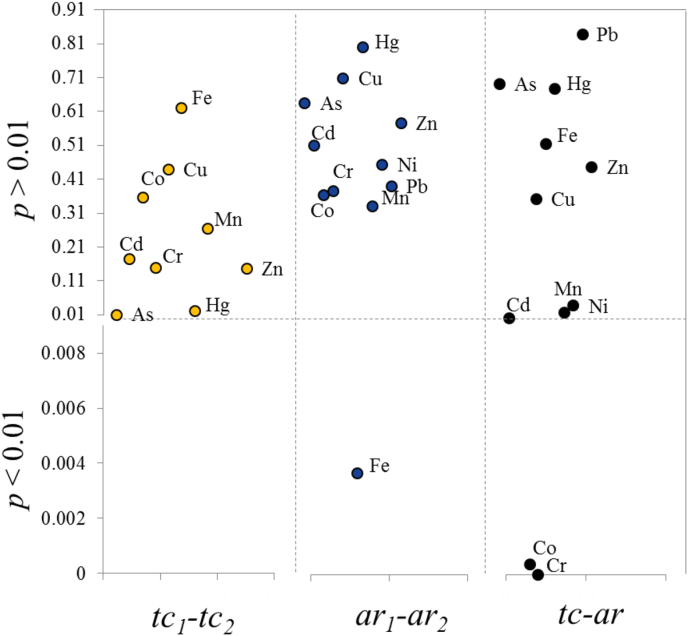
Results of the *t*-test (α = 0.01) for the total content determined using microwave digestion/spectroscopy (tc_1_) and *k*_0_-NAA (tc_2_), *aqua regia* extractable content determined with ISO 11466 (ar_1_) and ISO 12914 (ar_2_) and combined data for total content (tc) and *aqua regia* (ar).

**Fig. 2:**
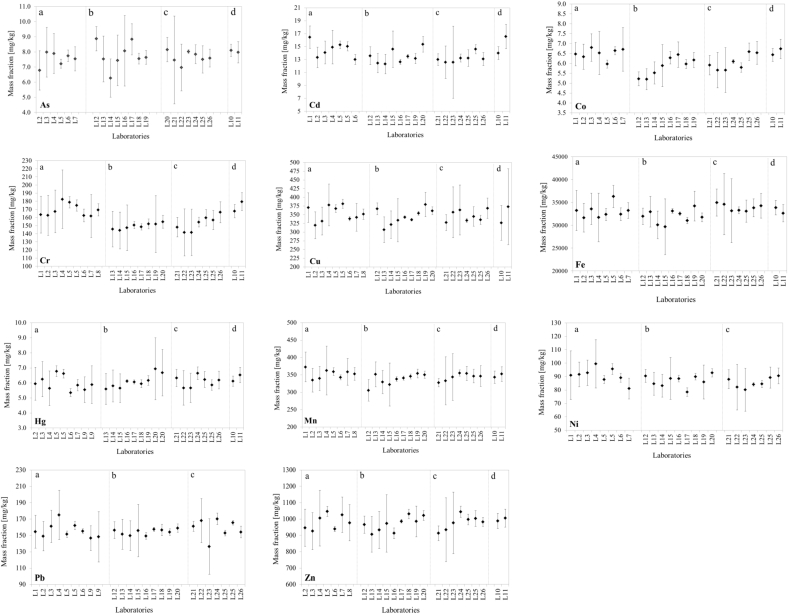
Mean values for each participating laboratory (◆) with the reported uncertainty budget as error bar (|). a) total content using microwave digestion combined to spectroscopic determination (tc_1_); b) *aqua regia* extractable content according to ISO 11466 (ar_1_); c) *aqua regia* extractable content according to ISO 12914 (ar_2_); d) total content on solid samples using *k*_*0*_-NAA (tc_2_).

The mean values of all results received for tc_1_ and tc_2_ and the corresponding between-measurements relative standard deviations (RSD, %) are presented in Table 2. RSDs for the tc_1_ and tc_2_ datasets were in comparable ranges: between 5% and 8% for all elements with the only exception of As being 13% for tc1 and between 1% and 10% for tc_2_ with the exception of 35% for As. The values reported in Table 2 show that there was no significant difference between the tc_1_ and tc_2_ results for each element, when the RSD is considered and this comparability was also reflected in the outcome of the *t*-test (*α*=0.01, Fig. 1), which reported no statistical difference between the tc_1_ and tc_2_ results (*p* > 0.01). These data show that methods involving microwave digestion followed by spectroscopic determination on average do not differ from those obtained by *k*_*0*_-NAA. However, by looking in details into the results provided by each participating laboratory, it emerged that the expanded uncertainties reported for the tc_1_ dataset ranged from 2% to 21% for L1–L9, with most of the uncertainties above 10% (Fig. 2). Laboratory L10 and L11 using *k*_*0*_-NAA instead reported lower uncertainty budgets, in all cases below 10% (mostly < 5%), with the only exceptions being Cd (12% for L10) and Cu (15% for L10 and 30% for L11). As detailed in Table 1a, Table 1ba and 1b, uncertainties were calculated taking into account a number of factors affecting the measurement results (*e.g.* sample digestion, repeated analyses, etc.), most of which are not applicable to *k*_*0*_-NAA being a solid-sample technique. As expected, the absence of a sample preparation step for this technique has a positive effect in reducing the overall uncertainty budget. Nonetheless, considering the non-significant difference between method datasets tc_1_ and tc_2_ as reported in Table 2, the microwave digestion methods used in this study can be considered reliable methods for the total content determination in such matrices, although higher uncertainties should be expected compared to the *k*_*0*_-NAA.

In Table 2, the guideline limit values for the analysed elements in Europe, U.S.A. and Canada, alongside with available literature data for China, Pakistan and India are also presented. The values found within this inter-laboratory comparison study reflected the typical values reported for heavy metals in municipal solid waste in Europe [24] and were below the limits set by the EU Directive 86/278/EEC [13] for Cd, Cu, Hg, Ni, Pb and Zn. They were also in line with data reported by Hargreaves et al. [10] for a number of sewage sludge materials (and compliant with USA and Canadian guideline limits, [25], [26]); slightly lower than the results reported by Cai et al. [27] and Wang et al. [28], for sewage sludge originating from WWTP in China, and by Usman et al. [6] for sewage sludge from Pakistan and India. This makes the material particularly suitable to be used widely by laboratories to check their method performance (*i.e.* reference material).

### 3.2 *Aqua regia* extractable content

As for the total content, the datasets for the *aqua regia* extractable content were all normally distributed and there were only a few outliers which were all retained for the final statistical evaluation, as no technical reason for their exclusion was identified. The average values of the results obtained for each element by all laboratories using ISO 11466 (L12–L20) and by the ones employing ISO 12914 procedure (L21–L26), named ar_1_ and ar_2_, were comparable with ratios ar_1_/ar_2_ between 94% and 103% (Table 3). The lower ratio (94%) was found for Fe suggesting that ISO 11466 could provide lower results (about 6%) than ISO 12914 procedure. A *t*-test (α = 0.01, Fig. 1) confirmed the significance of this small difference. However the 6% difference in the average values is covered by the standard deviation between laboratories. It is therefore concluded that also the extraction of Fe by the two *aqua regia* procedures leads to comparable results for this material. Regarding the measurement uncertainties as reported by each single laboratory (Fig. 2), they were found to be on average higher when ISO 11466 extraction method was used and up to 40% for elements such as As and Cd. This could possibly be related to the method itself which, although involving a higher amount of sample that should reduce the standard deviation of the measurements, it also involves more analytical steps each one bringing an uncertainty contribution to be accounted for in the total measurement uncertainty. To this adds the low concentration of some of the elements and in particular of As, Cd, Co and Hg which may have been more challenging to quantify for some methods with higher detection limits (typically ICP-OES).

### 3.3 Comparison total content vs *aqua regia* extractable content

The datasets for *aqua regia* obtained with the two methods (ar_1_ and ar_2_) as well as the total content ones (tc_1_ and tc_2_) were combined to give two datasets named ar and tc, respectively. The ratio ar/tc was calculated and resulted between 89 and 101%, with lowest values for Cd = 90%, Co = 91% and Cr = 89%. The *aqua regia* extractable content of these elements was in fact on average 10% lower compared to the total content. This difference was statistically significant for Co and Cr (*t*-test; p < 0.01) and most likely also for Cd (*t*-test; p = 0.01). As for Fe in the determination of the *aqua regia* extraction methods, this difference was covered by the RSDs of the corresponding average values, which were on average about 10% (specifically between 1.3% and 15.4%), showing that total content and *aqua regia* extractions in our study provided comparable results.

This conclusion was also supported by the assessment of the final certified values and expanded uncertainties for the material. The final certified values were assigned as *unweighted* means of the laboratories' means for total and *aqua regia* extractable content [29], whereas the expanded uncertainties were calculated as a combination of different contributions from the material heterogeneity, stability over time and inter-laboratory comparison study (*i.e.* standard deviation of laboratory means divided by √*n*, with *n* number of laboratories) with a coverage factor *k* = 2 corresponding to a 95% confidence level. In order to fulfil the traceability of the values, separate certified values for total and *aqua regia* extractable content for each analysed element were eventually assigned; however, these certified values overlap within their expanded uncertainties, which were on average 6.5%, with only Hg reporting the highest value of 10.8%.

### 3.4 Effect of the composition of the material on the results

The main composition of sewage sludge is shown in Table 4. The material presented low water content and activity (about 2.5% and 0.15, respectively) and a TC content of about 36% m/m which was almost entirely organic carbon, corresponding to a total organic matter (OM) of about 66% m/m [30]. Plant macronutrients were found in the following order Ca > P > Mg > K > Na, ranging from 31 g/kg to 1.8 g/kg. Aluminium was about 18 g/kg, most likely in the form of oxides [31], whereas silicate content was about 7% m/m (SiO_2_ = 73 ± 4 g/kg). The particle size analysis showed an average particle size of about 11.4 ± 0.4 μm (maximum particle size of about 250 μm). As discussed above, the total content of the elements with different extraction methods was found comparable to the *aqua regia* extractable content for all the analysed elements when taking into account the RSDs of the measurements. These results are in disagreement with previous studies, for instance by Ščančar et al. [18] where a difference, sometimes up to 50%, was found between total and *aqua regia* extractable content, especially of Cd, Cr and Ni. Ščančar et al. [18] attributed this difference to the presence of silicates or refractory aluminium, iron and manganese oxides which are not completely dissolved by *aqua regia* and to which these elements may be bound.

The sewage sludge used in this study had silicate (SiO_2_) content of about 7% m/m (Table 4) and an amount of refractory aluminium and iron oxides, theoretically Al_2_O_3_ and Fe_2_O_3_, below 10% (from Al and Fe values in Table 2, Table 3). These values are relatively low for sewage sludge and therefore probably not affecting the extraction of such elements by *aqua regia*. Perhaps the only noticeable effect is on Co and Cr, and to a certain extent Cd, for which *aqua regia* extractable content was found to be slightly lower than the total one (about 10%). Sastre et al. [20] also reported that for samples with high organic matter content (70%), milder solvents such as nitric acid could substitute HF in the extraction of Cd, Cu, Pb and Zn with negligible difference of the results. On the same line, other studies [32], [33] have demonstrated that elements such as Cr, Cu and Pb can be significantly related to the organic matter and thus are more readily available and more easily extractable. The high OM content of the sewage sludge material used in this study (about 66% m/m) may also have positively contributed to the extraction capacity of *aqua regia*.

A parameter also to be considered for the explanation of the results is the particle size of the material, on average about 11 μm, which has already been shown affecting the extraction of some elements in environmental matrices [34]. Smaller particles in fact may have facilitated the extraction of strongly bound elements by *aqua regia*, due to the greater surface area available for sample/solvent contact, bringing in this way the results close to the ones obtained with extractions employing stronger acids.

These considerations have to be seen not as a general rule but rather related to the specific chemical composition of the sewage sludge material used in this case study. Sewage sludge of different origin and composition may thus behave in a different way when undergoing extractions with *aqua regia* or more aggressive conditions.

## 4 Conclusions

Standardisation of operationally-defined procedures is of pivotal importance for the comparability of results. The use of quality control tools such as the certified reference materials is a key factor to additional assure the accuracy of the results.

In this work, within the characterisation study of a sewage sludge certified reference material, the performance of two *aqua regia* extraction ISO procedures and a number of total element content methods was compared. The results for the *aqua regia* extractable content obtained with the two ISO methods (ISO 11466:1995 and ISO 12914:2012) were comparable for all the analysed elements within their precision, meaning that there was no difference in the extraction efficiency of the two tested *aqua regia* methods. When compared to the total content ones through a *t*-test (α = 0.01), these values were also not significantly different for all elements but Co and Cr. This statistically significant difference was however technically not significant, as it was covered by the variation of the measurement results.

The comparability between total content and *aqua regia* extractable values is considered to be related to the intrinsic composition of the sewage sludge material, having low silicate and refractory oxide amount and high organic matter content as well as to the small particle size which may have enhanced the extraction of these elements by *aqua regia*, thus bringing these results in line with the results of analytical methods designed to obtain the total content.

## Figures and Tables

**Table 1a:** Total content methods. Summary of digestion conditions, instrumentations used and uncertainty budget approaches for all laboratories participating in the inter-laboratory comparison study. σ is the standard deviation of the measurements within laboratory; bias includes error from sample preparation.

Lab	Digestion method	Weight (g)	Instrumental analysis	Approach for uncertainty estimation
L1	5 mL HNO_3_/2 mL HCl/3 mL HF	1.0	*ICP-MS*: Perkin Elmer Elan 6000 DRC II (all elements) *ICP-OES*: Thermo Electron Atomscan 16 (Fe)	2σ + bias
L2	2 mL HNO_3_/6 mL HCl/2 mL HF/22 mL 4% H_3_BO_3_	0.3	*ICP-OES*: Perkin Elmer Optima 3000 DV (all elements) *CV-AAS*: Perkin Elmer FIMS 400 (Hg)	2σ + bias
L3	2 mL HNO_3_/6 mL HCl/2 mL HF/22 mL 4% H_3_BO_3_	0.3	*ICP-MS*: Perkin Elmer Elan DRC II (all elements) *CV-AAS*: Perkin Elmer FIMS 400 (Hg)	2σ + bias
L4	5 mL HNO_3_/3 mL HF	0.3	*ICP-SFMS*: Thermo Electron Corp, Cetac (all elements)	Ruth [35]
L5	4 mL HNO_3_/1 mL HCl/0.5 HF/5 mL H_2_O_2_	0.3 0.03	*FAAS*: Varian Spectra AA 110 (Cd, Cr, Cu, Fe, Mn, Ni, Pb, Zn) *ICP-MS*: Agilent 7500ce (As, Co) *CV-AAS*: Automatic Mercury Analyzer Model Hg-201 (Hg) DMA-80 Milestone (Hg)*	EURACHEM/CITAC guide [23]
L6	9.5 mL HNO_3_/0.5 mL HClO_4_/0.5 mL HF	0.25	*ICP-OES*: Spectroflame polychromator (all elements) *CV-AFS*: AMA Hydra II AF Gold (Hg)	2σ
L7	15 mL HNO_3_/1 mL HF	0.3	*ICP-MS*: Perkin Elmer DRC-e (As, Cd, Co) *ICP-OES*: Perkin Elmer Optima 3000 (Cr, Cu, Fe, Mn, Ni, Pb, Zn) *CV-AAS*: Perkin Elmer AA200 (Hg)	2σ
L8	20 mL HNO_3_/5 mL HF	0.1	*ICP-OES*: IRIS Intrepid II XDL (Cr, Cu, Mn, Zn)	EURACHEM/CITAC guide [23]
L9	15 mL HNO_3_/1 mL H_2_O_2_/2 mL HF	0.3	*ICP-MS*: PerkiElmer ELAN DRCe (Hg) *ICP-OES*: Optima 4300 DV Perkin Elmer (Pb)	2σ
L10	Solid sample	0.4	*k*_*0*_*-INAA*, HPGe detector (40%): As, Cd, Co, Cr, Cu, Fe, Hg, Mn, Zn	Robouch et al. [36]
L11	Solid sample	0.1	*k*_*0*_*-INAA*, HPGe detector (45%): As, Cd, Co, Cr, Cu, Fe, Hg, Mn, Zn	EURACHEM/CITAC guide [23]

**Table 1b:** *Aqua regia* extractable content methods (ISO 11499 [16] and ISO 12914 [17]). Summary of instrumentations used and uncertainty budget approaches for all laboratories participating in the inter-laboratory comparison study. σ is the standard deviation of the measurements within laboratory; bias includes error from sample preparation.

Lab	ISO 11499: 1995 [16]	Approach for uncertainty estimation
L12	*ICP-MS*: Perkin Elmer Elan 6000 DRC II (As, Cd, Co, Cr, Cu, Hg, Mn, Ni, Pb, Zn); *ICP-OES*: Thermo electron Atomscan 16 (Fe)	2σ + bias
L13	*ICP-OES*: Perkin Elmer Optima 3000 DV (all elements) *CV-AAS*: Perkin Elmer FIMS 400 (Hg)	2σ + bias
L14	*ICP-MS*: Perkin Elmer Elan DRC II (all elements) *CV-AAS*: Perkin Elmer FIMS 400 (Hg)	2σ + bias
L15	*ICP-SFMS*: Thermo Electron Corp, Cetac (all elements)	2σ
L16	*ICP-OES*: Spectroflame polychromator (all elements); *CV-AFS*: AMA Hydra II AF Gold (Hg)	2σ
L17	*ICP-MS*: Perkin Elmer DRC-e (As, Cd, Co); *ICP-OES*: Perkin Elmer Optima 3000 (Cr, Cu, Fe, Mn, Ni, Pb, Zn); *CV-AAS*: Perkin Elmer AA200 (Hg)	2σ
L18	*ICP-MS*: Agilent 7500ce (As, Cd, Co, Hg, Ni, Pb); *ICP-OES* Perkin Elmer Optima 3200RL (Cr, Cu, Fe, Mn, Zn)	2σ + bias
L19	*ICP-OES*: Perkin Elmer Optima 3000 DV (As, Cd, Co, Cr, Cu, Fe, Mn, Ni, Pb and Zn); *CV-AFS*: AMA Hydra II AF Gold (Hg)	EURACHEM/CITAC guide [23]
L20	*FAAS*: Varian Spectra AA 110 (Cd, Cr, Cu, Fe, Mn, Ni, Pb, Zn); *CV-AAS*: AMA Model Hg-201 (Hg)	2σ
Lab	ISO 12914: 2012 [17]	Approach for uncertainty estimation
L21	*ICP-SFMS*: Thermo Scientific Element XR (all elements)	2σ
L22	*ICP-OES*: OPTIMA 3000 XL (As, Cd, Co, Cr, Cu, Fe, Mn, Ni, Pb, Zn); *AAS*: AMA 254 (Hg)	σ
L23	*ICP-MS*: Agilent 7700 series (As, Cd, Co, Pb); *ICP-OES*: Varian 730-ES (Cr, Cu, Fe, Mn, Ni, Zn); *AFS*: Millenium Merlin (Hg)	σ
L24	*ICP-MS*: Agilent 7500ce (As, Cd, Co, Hg, Pb, Ni); *ICP-OES*: Perkin Elmer Optima 3200RL (Cr, Cu, Fe, Mn, Zn)	2σ
L25	*ICP-MS*: Agilent 7700× (all elements); *ICP-OES*: Thermo iCAP 6300 (As, Cd, Co, Cr, Cu, Fe, Pb, Mn, Ni, Zn); *CV-AFS*: Millennium System, PSA Analytical (Hg)	2σ
L26	*ICP-OES*: IRIS Advantage Thermo Elemental (As, Cd, Co, Cr, Cu, Fe, Pb, Mn, Ni, Zn); *CV-AFS*: AMA Hydra II AF Gold (Hg)	2σ + bias

*Direct mercury analyzer DMA-80 required no sample preparation.

**Table 2:** Mean values of all results obtained for tc_1_ (L1–L9) and tc_2_ (L10–L11). In parentheses, relative standard deviation of the measurements (%) and total number of replicate measurements (*n*).

Element	This study		Guideline limit values			Literature values	
	Total content (tc_1_)	*k*_*0*_*-*NAA (tc_2_)	EU [13]	U. S. A [25]	Canada [26]	China [27]	Pakistan and India [28]
As (mg/kg)	7.6 (13%, *n* = 36)	8.0 (35%, *n* = 12)	–	41	13	0.5–112	8–23
Cd (mg/kg)	14.6 (8%, *n* = 42)	15.3 (10%, *n* = 12)	20–40	–	5	–	2–9
Co (mg/kg)	6.5 (8%, *n* = 42)	6.6 (3%, *n* = 12)	–	39	3	–	–
Cr (mg/kg)	169 (5%, *n* = 54)	174 (5%, *n* = 12)	–	–	210	62–1844	66–1098
Cu (mg/kg)	354 (6%, *n* = 54)	350 (9%, *n* = 12)	100–1700	1500	400	120–2051	202
Fe (g/kg)	33.1 (5%, *n* = 48)	33.3 (3%, *n* = 12)	–	–	–	–	250
Hg (mg/kg)	6.0 (8%, *n* = 54)	6.3 (6%, *n* = 12)	16–25	17	0.8	–	7–32
Mn (mg/kg)	353 (6%, *n* = 48)	348 (3%, *n* = 12)	–	–	–	–	210
Ni (mg/kg)	91 (8%, *n* = 48)	–	300–400	420	62	–	12–596
Pb (mg/kg)	157 (8%, *n* = 54)	–	750–1200	300	150	14–134	26–154
Zn (mg/kg)	981 (7%, *n* = 42)	997 (1%, *n* = 12)	2500–4000	2800	700	850–6719	640

**Table 3:** Mean values of all results obtained for ar_1_ (L12–L20) and ar_2_ (L21–L26), with in parentheses the relative standard deviation of the measurements (%) and total number of replicate measurements (*n*), and detailed ratios ar_1_/ar_2_ and ar/tc where ar represents the combined datasets for *aqua regia* and tc represents the combined datasets for total content.

Element	*Aqua regia* extractable content (mg/kg)		Ratio (%)
	ISO 11466 (ar_1_)	ISO 12914 (ar_2_)	ar_1_/ar_2_
As (mg/kg)	7.8 (15%, *n* = 48)	7.6 (7%, *n* = 42)	103
Cd (mg/kg)	13.2 (7%, *n* = 48)	13.1 (5%, *n* = 42)	101
Co (mg/kg)	5.8 (9%, *n* = 48)	6.0 (5%, *n* = 42)	97
Cr (mg/kg)	149 (4%, *n* = 48)	152 (7%, *n* = 42)	98
Cu (mg/kg)	343 (7%, *n* = 54)	350 (5%, *n* = 42)	98
Fe (g/kg)	31.9 (5%, *n* = 54)	34.0 (4%, *n* = 42)	94
Hg (mg/kg)	6.0 (8%, *n* = 54)	6.1 (7%, *n* = 42)	98
Mn (mg/kg)	338 (5%, *n* = 54)	343 (4%, *n* = 42)	99
Ni (mg/kg)	87 (6%, *n* = 54)	85 (5%, *n* = 42)	102
Pb (mg/kg)	154 (3%, *n* = 54)	158 (8%, *n* = 42)	97
Zn (mg/kg)	968 (5%, *n* = 54)	975 (5%, *n* = 42)	99

**Table 4:** Main chemical and physical parameters (based on dry mass weight) for the sewage sludge material. *n* = number of bottles or unit tested.

Parameter	Average	Standard deviation	Unit
Water content (*n* = 6)	2.5	0.5	%
Water activity (*n* = 3)	0.15	0.01	%
TC (*n* = 2)	36.5	1.3	%
TIC (*n* = 2)	0.10	0.02	%
TOC (*n* = 2)	36.4	1.3	%
OM^a^	65.5	–	%
Al (*n* = 6)	18.5	0.2	g/kg
Ca (*n* = 6)	31.3	0.9	g/kg
K (*n* = 6)	2.9	0.1	g/kg
Mg (*n* = 6)	3.8	0.1	g/kg
Na (*n* = 6)	1.8	0.1	g/kg
P^b^ (*n* = 3)	9.5	0.4	g/kg
SiO_2_ (*n* = 3)	73	4	g/kg
Particle size (*n* = 3)	11.4	0.4	μm

a Organic matter (OM) was calculated multiplying TOC by a factor 1.8 [30].

b Determined as P_2_O_5_.
